# 

**Published:** 1875-09

**Authors:** 


Vol. 22. SEPTEMBER, 1875. No. 9.

TWENTY-SECOND YEAR.

### HALL'S

### Journal of Health.

"The serial, a portion of which appears in this number, entitled "OUR SUMMER AT MAPLEWOOD," has been duly copyrighted according to law."

PUBLISHED BY THE AMERICAN AND FOREIGN PUBLICATION CO., 137 EIGHTH ST., NEW YORK.

**Agents for the Trade:**

AMERICAN NEWS COMPANY, 121 NASSAU STREET.
NEW YORK NEWS COMPANY, 18 BEEKMAN STREET.

**Terms, Two Dollars a Year.**

